# Subsets of mononuclear phagocytes are enriched in the inflamed colons of patients with IBD

**DOI:** 10.1186/s12865-019-0322-z

**Published:** 2019-11-12

**Authors:** Hong Liu, Suryasarathi Dasgupta, Yu Fu, Brandi Bailey, Christian Roy, Eric Lightcap, Benjamin Faustin

**Affiliations:** 1 Immune-Oncology DDU, Takeda Pharmaceuticals, Cambridge, MA USA; 2 Immunology Unit, Takeda California Inc, San Diego, CA USA; 3 CNRS, UMR 5164, 33000 Bordeaux, France; 4 Immunology Discovery, Janssen Research and Development, San Diego, CA USA

**Keywords:** Inflammatory bowel disease, Crohn’s disease, Ulcerative colitis, Myeloid cells, Mononuclear phagocytes, Monocytes, Gut macrophages, Gut dendritic cells, GSEA, Cell type gene signature, ROC analysis

## Abstract

**Background:**

Myeloid cells, especially mononuclear phagocytes, which include monocytes, macrophages and dendritic cells (DC), play vital roles in innate immunity, and in the initiation and maintenance of adaptive immunity. While T cell-associated activation pathways and cytokines have been identified and evaluated in inflammatory bowel disease (IBD) patients (Neurath, Nat Rev Gastroenterol Hepatol 14:269–78, 1989), the role of mononuclear phagocytes are less understood. Recent reports support the crucial role of DC subsets in the development of acute colitis models (Arimura et al., Mucosal Immunol 10:957–70, 2017), and suggest they may contribute to the pathogenesis of ulcerative colitis (UC) by inducing Th1/Th2/Th17 responses (Matsuno et al., Inflamm Bowel Dis 23:1524–34, 2017).

**Results:**

We performed in silico analysis and evaluated the enrichment of immune cells, with a focus on mononuclear phagocytes in IBD patient colonic biopsies. Samples were from different gut locations, with different levels of disease severity, and with treatment response to current therapies. We observe enrichment of monocytes, M1 macrophages, activated DCs (aDCs) and plasmacytoid dendritic cells (pDCs) in inflamed tissues from various gut locations. This enrichment correlates with disease severity. Additionally, the same mononuclear phagocytes subsets are among the top enriched cell types in both infliximab and vedolizumab treatment non-responder samples. We further investigated the enrichment of selected DC and monocyte subsets based on gene signatures derived from a DC- and monocyte-focused single cell RNA-seq (scRNA-seq) study (Villani et al., Science 356:eaah4573, 2017), and verified enrichment in both inflamed tissues and those with treatment resistance. Moreover, we validated an increased mononuclear phagocyte subset abundance in a Dextran Sulphate Sodium (DSS) induced colitis model in C57Bl/6 mice representative of chronic inflammation.

**Conclusions:**

We conducted an extensive analysis of immune cell populations in IBD patient colonic samples and identified enriched subsets of monocytes, macrophages and dendritic cells in inflamed tissues. Understanding how they interact with other immune cells and other cells in the colonic microenvironment such as epithelial and stromal cells will help us to delineate disease pathogenesis.

## Background

Inflammatory bowel disease, with Crohn’s disease (CD) and ulcerative colitis as two major subsets, is a complex chronic inflammatory disorder of the human gastrointestinal tract with unknown causes. There is a large unmet medical need for novel therapeutic approaches as many patients do not respond to clinically approved drugs, including TNF blockers (e.g. infliximab) and anti-integrin (vedolizumab) therapies and frequently relapse [1]. For CD, approximately 40% of patients do not respond to the initial anti-TNF therapy and are also unlikely to benefit from another TNF antagonists [2]. Additionally, 30–50% of patients eventually become unresponsive to treatment [3, 4]. Similarly, the primary response rate for vedolizumab ranges between 49 and 64% in CD and 43–57% in UC [5]; and 40–50% of patients lose response over time [6]. The molecular mechanisms underlying treatment response, resistance or relapse are unknown.

Gut inflammation occurring in patients with IBD has traditionally been associated with exaggerated Th1 or Th2 (and possibly Th17) response [7, 8]. Specifically, CD has been associated with Th1 cell lineage and UC is characteristic with Th2 immune response. Recent evidence suggests a contribution from the innate immunity which have linked innate lymphoid cells (ILC) to IBD pathogenesis [9]. Mononuclear phagocytes, comprising professional antigen presenting cells like macrophages and dendritic cells among others, are known for their important role in innate and adaptive immune response to infection and tissue injury [10]. They possess multiple pathogen and danger sensing receptors along with antigen processing and presentation capabilities. Mounting evidence indicates gut-specific mononuclear phagocytes and their characteristic participation in murine models of colitis. Although critical to translational research, the knowledge about their human counterparts is very limited. Initial studies focused on circulating DCs and identified activation in IBD patients compared to non-IBD controls [11, 12]. Further, colonic DCs were shown to enhance TLRs (Toll-like receptors) in IBD. In CD, DCs were found to exacerbate the expression of CD40, IL12 and IL6 [13]. Importantly, mature myeloid CCR7+ DCs were reported to cluster with proliferating T cells in CD patient colons suggesting a direct role in modulating T cell dependent chronic pathology [14]. The role of mononuclear phagocytes in polarizing T cells was further confirmed when CD163 lo Macrophages from colonic lamina propria (LP) of CD patients, were shown to induce polarization of Th17 T cells ex vivo. These cells from CD patients, resembling M1-like macrophages, polarized T cells at a better rate than similar cells from non-inflamed region or from non-IBD patients [15].

In addition to their role in pathogenesis, recent studies indicated a potential linkage between myeloid cells and treatment non-responsiveness with biologics. CD14+ macrophages, which produce IL-23 and activate intestinal apoptosis-resistant TNFR2 + IL23R+ T cells, were found to be significantly more present in non-responders prior to anti-TNF treatment [4]. Vedolizumab (an anti-α4β7 integrin antibody) treatment was associated with substantial effects on the innate immune system including changes in macrophage populations and pronounced alterations in the expression of microbial sensing molecules, chemoattraction and innate effector response [16]. Furthermore, an in silico analysis suggested an increase in inflammatory macrophages in pretreatment intestinal biopsies from anti-TNF non-responding individuals [17]. In addition, mononuclear phagocytes function is highly associated with more severe disease outcomes observed in treatment refractory patients. Thus, improved knowledge of mononuclear phagocyte function and regulation in IBD may illuminate novel therapeutic strategies [18].

The mechanistic role of these cells in murine models of colitis is important and consistent with patient-derived data [19, 20]. For example, it has been reported that monocyte/macrophage mobilization is required for colitis development [21], and that LP-resident CD169+ macrophages play a pivotal role in the progression of mucosal injury by producing CCL8 [22]. In addition, pDCs were also found to play a crucial role in the development of acute colitis through the control of the intestinal inflammatory response [23].

Recently, computational techniques to decipher cellular content directly from genomic profiles of cellular mixture have been developed. These techniques have been applied to characterize tumor infiltrating leukocytes in human tumors to facilitate personalized cancer therapy [24]. Similar approaches can be applied to investigate and delineate the immune cell landscape in inflamed tissues, including IBD colon biopsies. In general, there are two main types of in silico methods. One is to deconvolute the complete cellular composition and the other is to assess the enrichments of individual cell types in the mixtures [24, 25]. For gene set enrichment analysis, GSEA [26] is a powerful tool to test whether a gene set shows statistically significant differences between two biological groups. Similarly, GSVA and ssGSEA could be applied to assess the enrichment of a gene set in the top of a ranked gene expression profile for an individual sample [27]. One of the most important factors is to use a gene set/gene signature that is specific to the cell type or state in question. To dissect mononuclear phagocyte populations in IBD and their impact in disease prognosis and treatment response, we used gene signatures generated from xCell, which were processed from over a thousand human cell type transcriptomes derived from various sources. xCell employs ssGSEA analysis, combining a curve fitting approach for linear comparison of cell types and a novel spillover compensation technique for separating closely related cell types [25]. For myeloid specific gene signatures, we focus on a set of six recently described DC subtypes and four Monocyte subtypes [28].

Here, we describe the IBD inflamed colon immune landscapes, with the notable enrichment of monocytes, M1 macrophages, aDCs and pDCs myeloid cells. The composition of different cells in inflamed tissue will provide new insights of pathways involved in disease, and delineate the key cellular drivers involved in IBD pathogenesis to open new therapeutic avenues.

## Results

### Several mononuclear phagocyte subsets are enriched in IBD inflamed colons

**Fig. 1:**
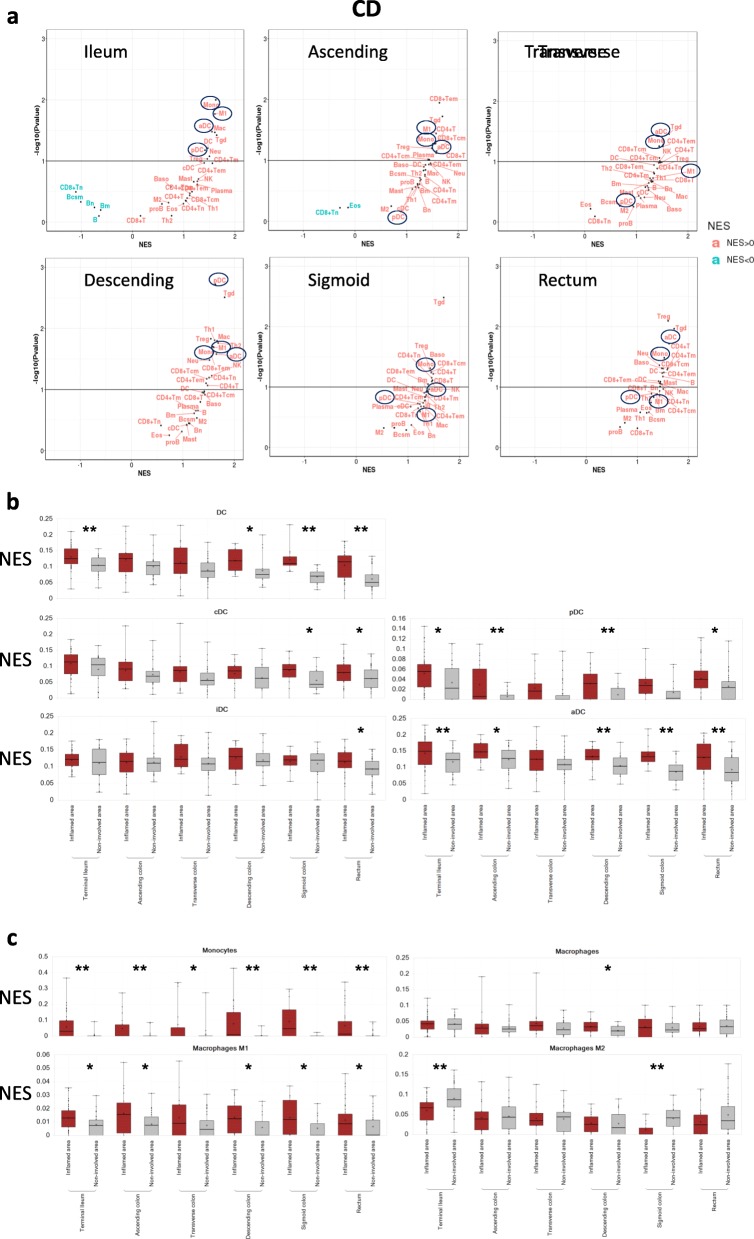
Immune cell enrichment in different gut locations of CD patients. **a** Cell types enriched in inflamed vs. non-inflamed tissue. Myeloid cell enrichment scores in inflamed and non-inflamed tissue from different gut sites: (**b**) DC subsets; (**c**) Monocyte and macrophage subsets. NES: normalized enrichment score. * *P* value < 0.05, ** *P* value < 0.01

GSEA analysis comparing inflamed vs. non-inflamed tissue was performed to evaluate immune cell enrichment, including both innate and adaptive cell types, at different Crohn’s colonic locations in the colon. Overall, we observed immune cell enrichment at all locations of inflamed colon (Fig. 1a). Mononuclear phagocytes, especially monocyte, M1, aDC and pDC were among the most significantly enriched cell types in the ileum and descending colon. As a positive cellular control, distal colon was also enriched in Th1, Th2, CD4+ or CD8+ tissue effector memory cells amongst others. The enrichment of mononuclear phagocytes was confirmed by single sample enrichment analysis (ssGSEA) which showed that those myeloid cells were significantly enriched in inflamed colon from most locations (Fig. 1b, c). Among all the dendritic cell types, the enrichment of aDC, pDC and DC are significantly different between inflamed and non-inflamed colons in most locations. Among all the macrophage and monocyte signatures, the enrichment of monocytes and M1 are significantly different between inflamed and non-inflamed colons in most locations. Interestingly, M2 macrophages shows an opposing enrichment and are significantly enriched in non-inflamed ileum and sigmoid tissue.

**Fig. 2:**
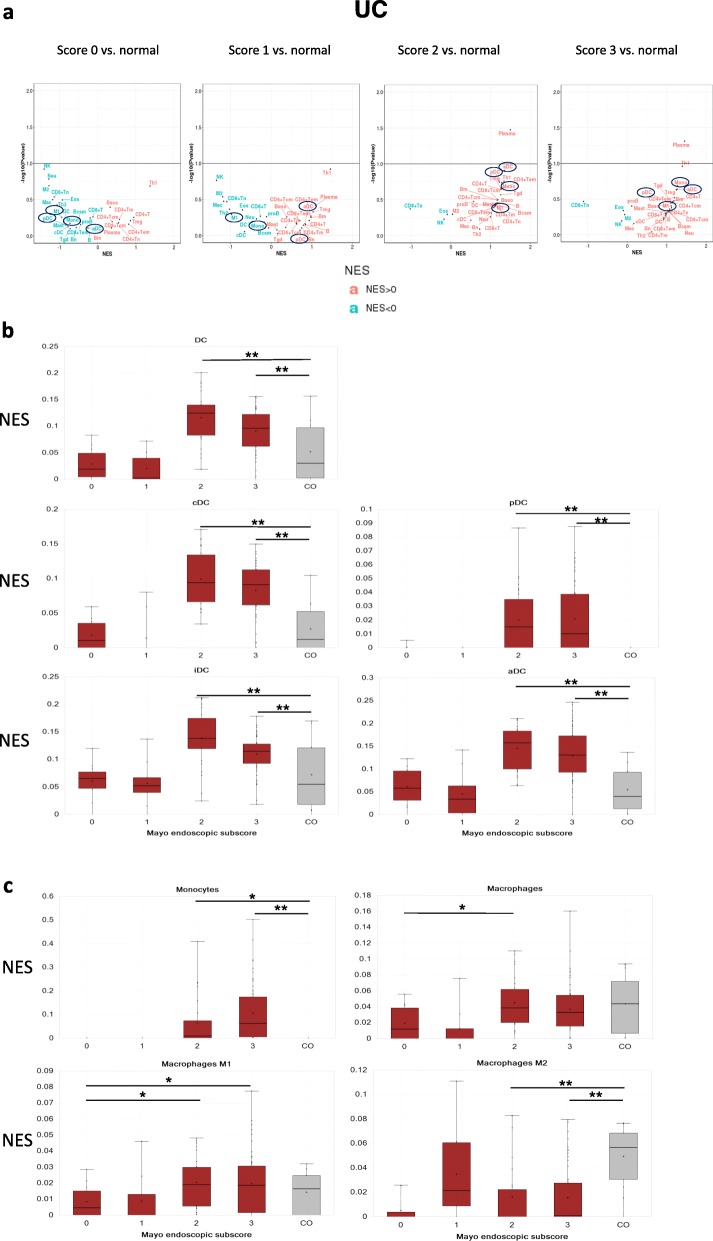
Immune cell enrichment at different pathological stages of UC patients. **a** Cell types enriched in patients with different mayo endoscopic scores vs. normal controls. Myeloid cell enrichment scores in samples with different mayo endoscopic scores: (**b**) DC subsets; (**c**) Monocyte and macrophage subsets. Score 0–3: Mayo endoscopic sub-scores. Normal control (CO): colonic mucosal biopsy from normal individual. NES: normalized enrichment score. * *P* value < 0.05, ** *P* value < 0.01

In a separate UC data set with endoscopic scores, mononuclear phagocytes are enriched in advanced biopsies with mayo endoscopic scores of 2 or 3, comparing to normal control (Fig. 2a). ssGSEA confirmed that the enrichment scores of all DC subsets and monocyte in advanced patient samples are significantly higher than that of normal control (Fig. 2b, c). Macrophage and M1 macrophage enrichments show no significant difference between advanced samples and normal controls. However, their enrichment scores are significantly higher in advanced samples than those in samples with lower score (Fig. 2c). On the other side, M2 macrophages are significantly enriched in the control colon comparing to that in those severely diseased colon tissues. Similar to CD samples, we again observed that some T cell subset enrichments correlate with disease severity. One such subset is Th1, which is known to produce IFN-γ and play an important role in gut pathology. Overall, these data suggest that myeloid cell populations, including M1 macrophages and aDC, accumulate in inflamed regions and are associated with disease severity.

### Several mononuclear phagocyte subsets are enriched in infliximab and vedolizumab non-responding biopsies

**Fig. 3:**
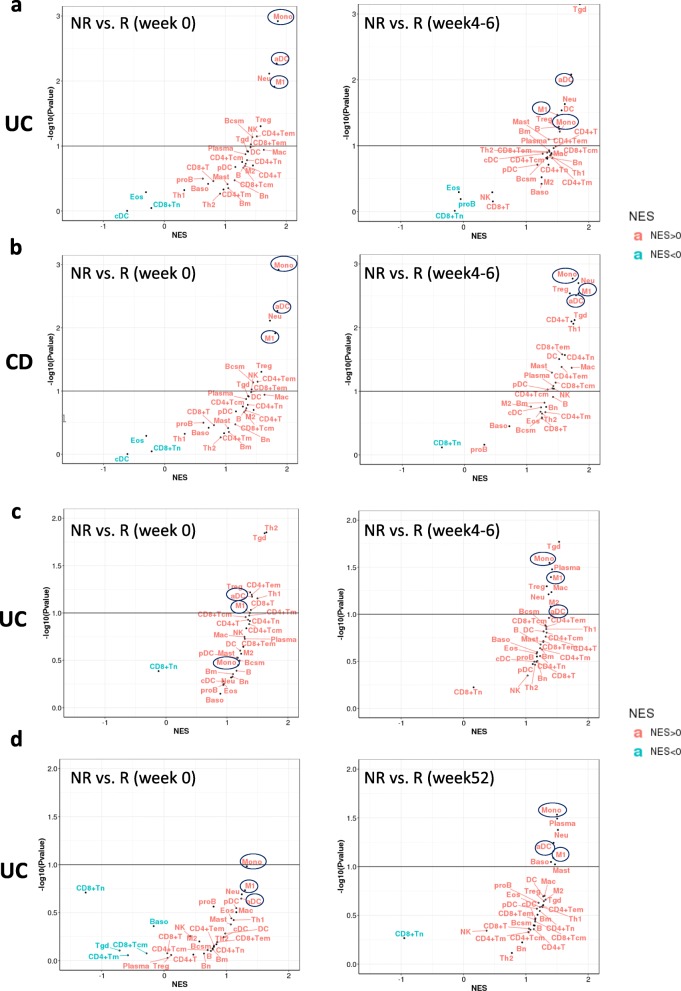
Cell type enrichment in response to biological treatment in IBD patient. **a** Cell type enriched in non-responder vs. responder to infliximab in UC (GSE16879). **b** Cell type enriched in non-responder vs. responder to infliximab in CD (GSE16879). **c** Cell type enriched in non-responder vs. responder to infliximab in UC (GSE73661). **d** Cell type enriched in non-responder vs. responder to vedolizumab in UC (GSE73661). NES: normalized enrichment score. NR: non-responder; R: responder

Current biological treatments for IBD, while effective, are not optimal. Although more than half of patients respond to treatment, many lose response or become resistant over time [29]. The mechanisms which lead to loss of response are not known. A better understanding of these mechanisms could enable novel therapeutic target identification. We determined immune cell type enrichments in samples from responding and non-responding patients including both pre- and post- treatment conditions. Out of the three independent infliximab treatment data sets, two strongly support mononuclear phagocytes (aDC, Monocytes, M1) as being among the top enriched cell types in the non-responding patients in both pre- and post- treatment samples (Fig. 3a, b), while the other dataset shows enrichment in the post treatment samples (Fig. 3c). Similarly, in vedolizumab-treated samples (Fig. 3d), there is a clear mononuclear phagocytes enrichment in non-responders before treatment, and they are further highly enriched after treatment. In order to investigate whether non-responders have more severe disease, we investigated the disease scores in pre-treatment samples for both infliximab and vedolizumab where data are available. There is no difference between responder vs. non-responder disease scores, which indicates that disease severity is not a factor that leads to treatment resistance.

To evaluate the prediction performance of each cell type for treatment non-response, we used ROC to measure the true-positive rates against the false positive rates at various thresholds of prediction scores and calculated the AUC score. Table 1 lists the top 10 immune cells sorted by AUC score. All 10 cell types predict non-responders better than random (i.e. all AUC scores > 0.5). Scores are higher in infliximab response prediction, especially for CD patients. Among the top ranked immune cell types, macrophage M1, monocytes and aDC are frequently found with higher AUC scores, which suggest higher prediction power.

**Table 1:** Top 10 cell types and their AUC scores for treatment non-response

Infliximimab Non-Response						Vedolizumab Non-Response	
UC (GSE16879)		CD (GSE16879)		UC (GSE73661)		UC (GSE73661)	
Cell type	AUC	Cell type	AUC	Cell type	AUC	Cell type	AUC
Macrophages.M1	0.84	Monocytes	0.99	Tregs	0.84	Monocytes	0.7
aDC	0.84	Neutrophils	0.98	Tgd.cells	0.83	Mast.cells	0.67
Tregs	0.81	Macrophages.M1	0.83	CD4.naive. T.cells	0.82	Macrophages.M1	0.64
Tgd.cells	0.79	aDC	0.83	Th2.cells	0.8	Macrophages	0.59
Monocytes	0.78	pDC	0.81	CD8.Tcm	0.77	Th1.cells	0.58
CD8.Tcm	0.77	CD8.Tem	0.77	CD4.Tem	0.77	DC	0.58
Th2.cells	0.74	Macrophages	0.75	CD4.memory. T.cells	0.76	Neutrophils	0.58
CD4.T.cells	0.73	B.cells	0.73	Macrophages.M1	0.75	Th2.cells	0.58
Neutrophils	0.73	CD4.Tcm	0.72	CD4.Tcm	0.74	aDC	0.58
CD4.Tcm	0.71	Tregs	0.71	aDC	0.73	Basophils	0.55

### DC and monocytes are enriched in diseased and treatment-resistant samples

**Fig. 4:**
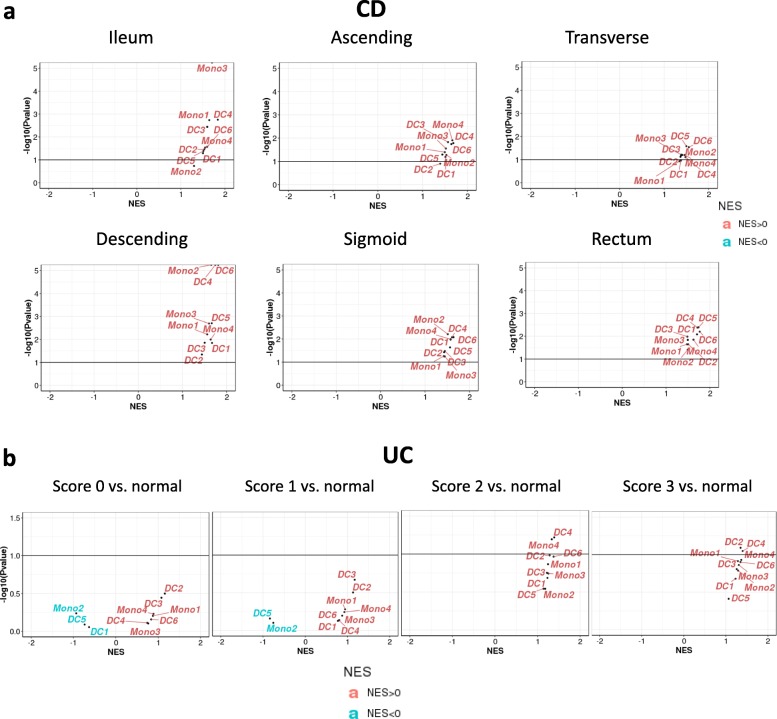
DC, monocyte subsets enrichment. **a** Cell subset enrichment in inflamed vs. non-inflamed tissue from different gut locations of CD patients. **b** Cell subset enrichment in UC samples with different mayo endoscopic scores vs. normal controls. Score 0–3: Mayo endoscopic sub-scores. Normal: colonic mucosal biopsy from normal individual. NES: normalized enrichment score

We measured the enrichment of different myeloid subsets in IBD colon biopsies using the adopted ten gene signatures representing 6 DCs (DC1 to DC6) and 4 monocytes (Mono1 to Mono4) from Villani et al. [28], and assessed their enrichment in the datasets discussed above. For the DC subsets, conventional DC1, known for antigen cross presentation to CD8 T cells, was defined by CLEC9A instead of the classical surface marker CD141. DC2 and DC3 were both defined by the myeloid cell marker CD1C, but like DC1 shared a common conventional DC (cDC) progenitor. However, DC2 and DC3 differed from one another as DC3 can also originate from monocyte precursors (Mono1, CD14+ classical monocytes). DC4 was defined by absence of DC1/DC2/DC3 phenotyping markers CD141 and CD1C while retaining the generic myeloid marker CD11C. DC4 was not connected to the common cDC progenitor but instead to Mono2 (CD16+ classical monocytes). DC6 were the classical pDC, while the new DC5 showed features of both conventional and plasmacytoid DC. DC5 was marked by AXL + SIGLEC6+. Finally, they reported two new monocyte subsets: Mono3 and Mono4. Our results show that these DC and monocyte signatures are enriched in CD patient inflamed vs non-inflamed colon samples (Fig. 4a). Specifically, the Mono3 signature is highly enriched in the ileum, and Mono2, DC4 and DC6 signatures are highly enriched in the descending colon. For the UC samples with different disease scores, DC2, DC4, and Mono4 are highly enriched in the severe disease (Fig. 4b).

**Fig. 5:**
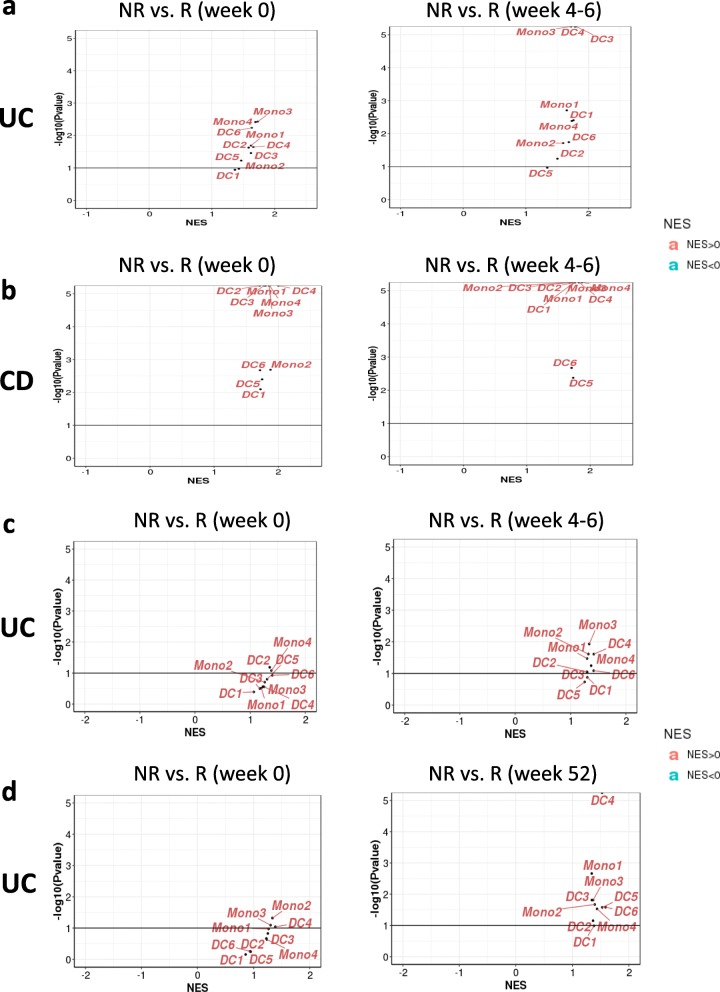
DC, monocyte subsets enrichment in response to biological treatment in IBD patient. **a** Cell type enriched in non-responder vs. responder to infliximab in UC (GSE16879). **b** Cell type enriched in non-responder vs. responder to infliximab in CD (GSE16879). **c** Cell type enriched in non-responder vs. responder to infliximab in UC (GSE73661). **d** Cell type enriched in non-responder vs. responder to vedolizumab in UC (GSE73661). NES: normalized enrichment score. NR: non-responder; R: responder

We further assessed the ten gene signatures in the patient samples with treatment response vs. resistance. In samples with infliximab treatment, most of those gene signatures are enriched in non-responders, and DC1 and DC5 are slightly less enriched (Fig. 5a, b, c). In samples with vedolizumab treatment, while most signatures are enriched in non-responders after treatment, DC4 is the most enriched (Fig. 5d).

**Fig. 6:**
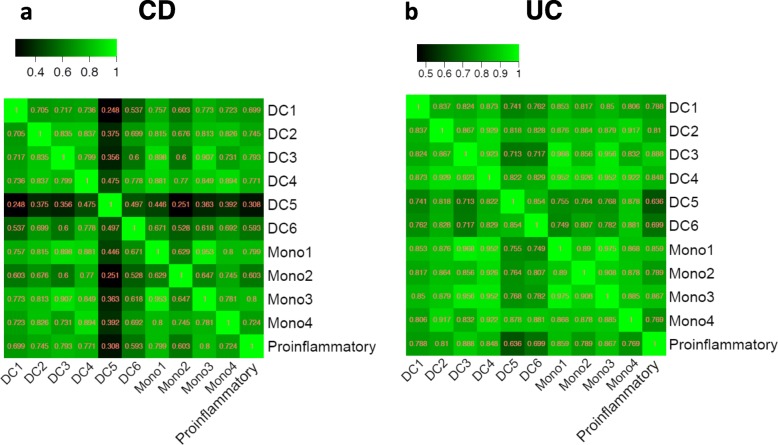
Correlation coefficient between DC, monocyte subsets and proinflammatory gene signatures in CD (**a**) and UC (**b**) patients

To evaluate the connection of DC and monocyte subtypes and explore their function, we determined the correlation between subset expression in IBD data sets (Fig. 6). Most subsets, with the exception of DC5 and potentially DC6, show a high correlation with each other in both CD and UC patients (CD ρ > 0.6, UC ρ > 0.8). Most cell type expressions are strongly correlated (ρ > 0.5) with a panel of proinflammatory gene signature (IL1B, IL1A, IL23, IL12A, IL12B, TNFA, IL6, IL8, IL18, IFNA1, IFNB1), except for DC5 in CD.

Next, we assessed the prediction performance of each DC, monocyte subset for treatment non-response. ROC was used to measure the true-positive rates against the false positive rates at various thresholds of prediction scores (Additional file 1: Figure S1). All ROC AUC scores are above 0.5 (random). Similarly, we found that the scores are higher in infliximab treatment prediction, especially for CD patients (Additional file 1: Figure S1 d).

### IBD tissue microenvironment: the myeloid footprint

**Fig. 7:**
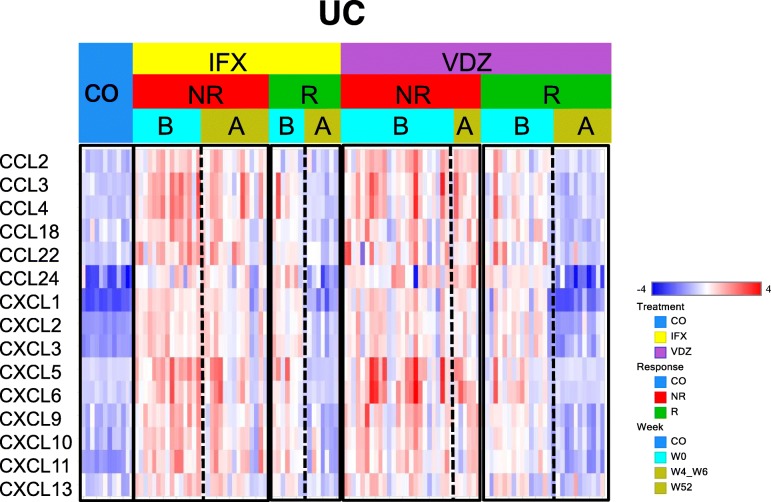
Chemokines retain high expression in non-responders after treatment (GSE73661). IFX: infliximab; VDZ: vedolizumab; NR: non-responder; R: responder; B: before treatment (Week 0); A: after treatment (Week 4–6 for infliximab; Week 52 for vedolizumab)

Chemokines are chemotactic cytokines that control immune cell migratory patterns and positioning, and are critical for their development and homeostasis [30]. We evaluated chemokine expression and identified a set whose expression was up-regulated in IBD colon biopsies, and the up-regulation pattern was retained only in non-responders after treatment (Fig. 7 and Additional file 1: Figure S2 a). Among those cytokines, CXCL1/2/3/5/6, CCL2/3/4/24 are reported to be involved in myeloid cell trafficking [30]. It provides additional evidence for the enrichment of myeloid subsets in inflamed and treatment resistant samples that we observe. Other chemokines (CXCL9/10/11/13, CCL18/22) with a similar pattern are involved in Th1/Th2 response and migration. This is consistent with T cell involvement in disease pathogenesis. To further quantify the chemokines involved in myeloid cell trafficking [30], we examined their median expression in related data sets. Shown in Additional file 1: Figure S2 b, c, there is a distinct up-regulation pattern of those chemokines in non-responder vs. responder population, in both pre- and post- treatment groups for either infliximab or vedolizumab.

In order to delineate the cell types in the colon tissue microenvironment contributing to dys-regulation of those myeloid chemokines, we leveraged a recent scRNA-seq data from human stromal cells [31]. CXCL1 and CXCL2 was up-regulated while CCL2 was not. Expression of other chemokines are either rare (CXCL3/5/6, CCL3/4) or not detectable (CCL24) (Additional file 1: Figure S3). These observations suggest that colonic stromal cells may contribute to the expression of CXCL1/2, while other cells—such as epithelial cells—are likely contributing to the chemokine dys-regulation observed in mixed colon tissues.

### Mononuclear phagocytes are enriched in the chronic DSS colitis model

In vivo murine model of colitis, amongst other features, provide a platform to enumerate mononuclear phagocytes in situ during inflammation development. In addition, advancements in transcriptomics and other technologies allow the selective ablation of particular mononuclear phagocytes and understand their roles in pathology using these murine models of disease. However, surface proteins are not necessarily shared between murine and human cells. Hence, we focused on standard mononuclear phagocyte phenotype in the mouse colon relying on their broad functional classification. Myeloid cell isolation procedures and subsequent phenotype analysis by Flow Cytometry needed to divide them into two fractions: intraepithelial lymphocyte fraction (IEL) and lamina propria fraction. Both fractions taken together, allowed us to consider myeloid subset changes in the entire colon.

**Fig. 8:**
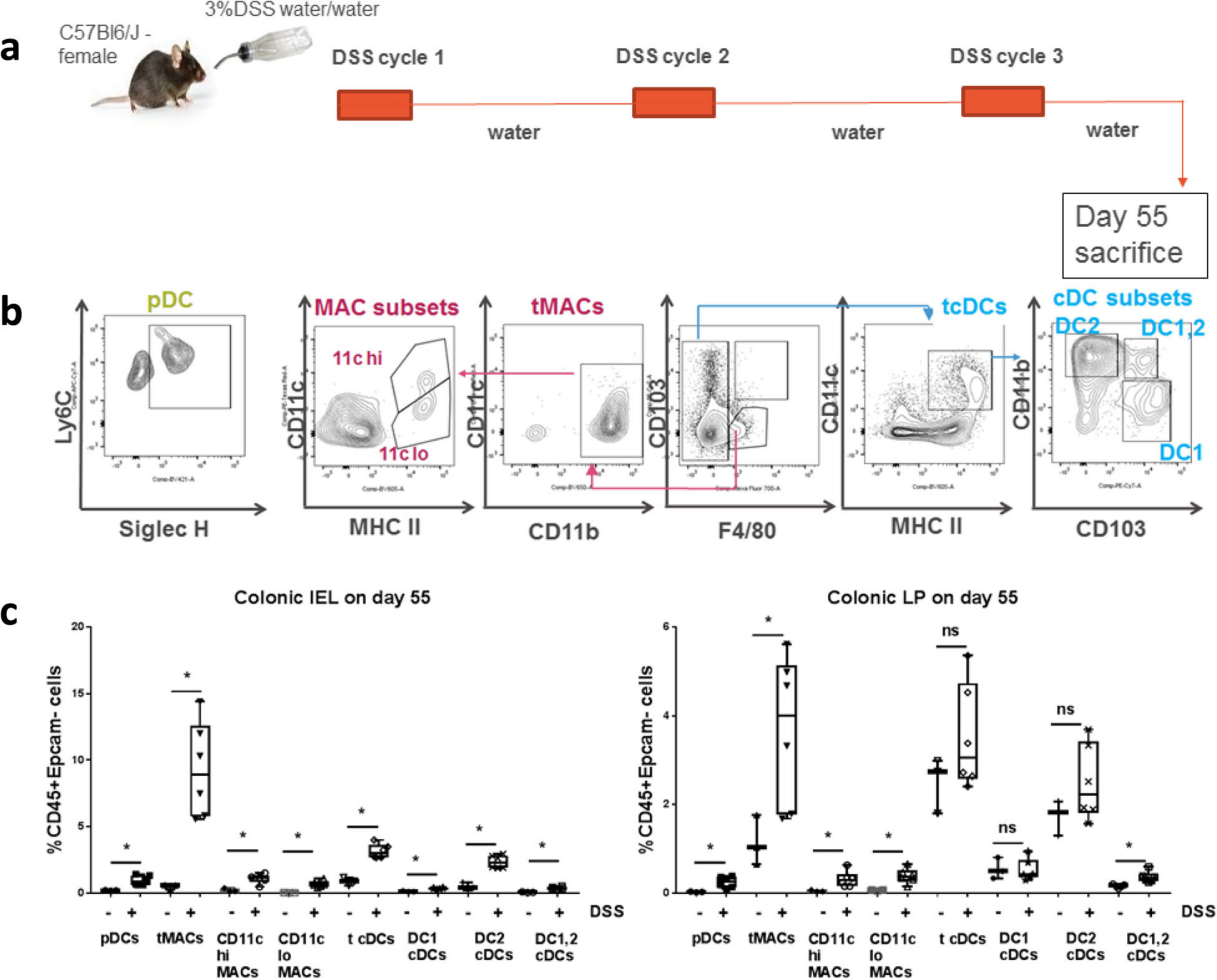
Augmentation of macrophages and dendritic cell subsets in mouse colon following chronic DSS stimulation. **a** Schematic representation of chronic DSS experiment. **b** Flow Cytometry gating for macrophages and dendritic cell subsets studied (shown for colonic LP). For pDCs, the pre-gating was Viable>CD45 + Epcam- > CD3-CD19- > CD11c + CD11b- > Ly6c + B220+ events; For macrophages and conventional dendritic cells, the pre-gating was Viable>CD45 + Epcam- > CD3-CD19->. Dendritic cells studied are total conventional dendritic cells or tcDCs (CD11c + MHCII+F4/80-); cDC subsets: DC1 (CD103 + CD11b-), DC2 (CD103-CD11b+) and double positive DC1, 2 (CD103 + CD11b+); and pDCs (SiglecH+Ly6c + B220 + CD11b-CD11c+). Macrophages studied are total macrophages or tMACs (CD103-F4/80 + CD11b+); CD11chi MHCII+ subset and CD11clo MHCII+ ones. **c** Changes in macrophage and dendritic cell frequencies (reported as amongst total hematopoietic cells) following chronic DSS stimulation in colonic IEL (left panel) and colonic LP (right panel). Each dot in the boxes represents each mouse which were untreated with DSS (*n* = 3) or fed DSS water for 3 cycles (*n* = 6). Non-parametric analysis (Mann-Whitney test) was used to evaluate significance. * means < 0.05

The DSS induced colitis model depicts chronic pathology to a greater extent than the often used acute DSS model (Fig. 8). The overall pathology initiation is similar between the two models, as both display epithelial barrier injury exposing immune cells to luminal pathogens. However, the cellular activity is reported to be different. Using the chronic DSS model, we found inflammation expanded total and potentially activated MHCII+CD11c hi/lo macrophage subsets, in both IEL and LP fractions. Total cDC and all DC subsets, including DC1 and DC2, were more enhanced in the IEL fraction than in the LP fraction. Interestingly, we also found that cDCs, with features of both DC1 and DC2, were enhanced in both fractions. DC2 is the myeloid DC subset in the mouse, expressing CD11b, and well known for T cell activation. DC1 s expressing CD103 have been shown to generate and sustain Tregs and cross present antigen to CD8 T cells. Recently, murine DC1 were also shown to interact with intestinal epithelial cells, thereby helping them maintain barrier integrity in an acute DSS colitis murine model [32]. Lastly, we observed inflammation-induced expansion of pDCs which have been reported to be pathogenic in an acute DSS colitis model [2], but not in more spontaneous IBD models [33] and induced into tolerogenic cells by commensal bacterial antigen thereby conferring protection [34].

## Discussion

The current manuscript performed systematic in silico analyses to de-convolute 31 immune cell types, with a focus on MNPs, in colonic mucosal biopsies in multiple publicly available data sets, which include both CD and UC, as well as different locations of the inflamed gut. Besides comparing inflamed vs. non-inflamed tissues, in-depth analyses were concentrated on treatment response mechanisms, for both infliximab and vedolizumab. It outlines an emerging picture - elevated mononuclear phagocyte subsets in inflamed colonic mucosal biopsies, and the pattern persisting only in non-responders after either infliximab or vedolizumab treatment. Further, we observed a correlated elevated expression of chemokine subset, which plays a role in myeloid cell function and contribute to generate and sustain inflammatory pathology. More importantly, in addition to in silico analysis, we also evaluated the levels of several macrophage and DC subsets; and confirmed their elevated abundance in a chronic DSS colitis model.

Gene signatures are gene sets that are coordinately regulated under specific biological conditions or across multiple biological states. Use of gene sets improves tolerance to noise and variability between samples, batches, or platforms, and can lead to novel interpretations of large-scale genomic data [35]. Gene signatures representing different immune cells are used to deconvolute tumor microenvironment immune contexture [24]. We started our first set of analysis by evaluating the gene signatures of 31 immune cell types for their enrichment in IBD patient colon biopsies. The clear mononuclear phagocyte enrichment pattern encouraged us to perform a second validation analysis using ten myeloid subset gene signatures derived from a scRNA-seq study [28].

The scRNA-seq approach focused on subsets of DCs and monocytes and thereby was more resolved but limited in scope. Since HLADR+lin- events were sorted prior to analysis, in-depth knowledge on circulating antigen presenting cells were generated. However, since macrophages are absent in blood, monocyte precursors of tissue macrophages were probed in the context of IBD. Thus, the first set of analysis allowed us to generate data on M1 and M2 polarized macrophage gene signatures both in CD and UC samples, where the potentially pro-inflammatory and thus pathogenic M1 subset was enhanced. In contrast, the potentially anti-inflammatory and thus protective M2 subset was diminished with severity. In terms of DC subsets, the scRNA-seq data stratified DCs based on non-inflammatory healthy situation. Thus, the first set of analysis could additionally capture DC subsets which can be a byproduct of inflammation (iDCs) or activation (aDCs). Indeed, aDCs were found to be highly enriched with disease severity in UC and CD, and also with treatment non-responsiveness across the spectrum studied. Though the exact surface phenotype for aDCs remains a question, like M1 and M2, aDC’s gene signature may be the only link in realizing their potential. pDCs or DC6 comes out as one of the links in the two analyses. Interestingly, along with DC5, which shares some features of pDCs, they are highly enriched specifically in the ileum and distal colon of CD patients (Figs. 1 and 4). Additionally, in drug non-responsiveness, pDCs came in the selected cell types to be enriched with infliximab non-responders in CD as per the first analysis. While in the scRNA-seq gene signature-based analysis, pDCs or DC6 were enriched in most cases of non-responsiveness studied. In case of cells like pDCs, for which a surface phenotype is comparatively better studied, scRNA-seq gene signature-based analysis may be effective.

Our murine model of chronic colitis showed some commonalities with reports in recent IBD literature. Human CD11c expressing intestinal macrophages were recently shown to produce major pro-inflammatory cytokine IL1b following LPS stimulation and were found to be enhanced in IBD patients’ colon [36]. Interestingly for DC2, the human analogue of CD1c-expressing DC2 and not their counterpart DC1 (expressing CD141), have been recently shown to co-express the TGFβ catalyzing integrin which is enhanced in intestine of IBD patients [37]. It was further shown that human DCs enhanced FOXP3 + CD4 T cells were in an integrin-dependent manner thereby emphasizing their importance in IBD. Thus, DC2 s present a case of mononuclear phagocytes with potentially translational value in IBD research. Our observation of the expansion of CD103 + CD11b + cDC subset has also been associated with Muc2 deficiency in mice and spreading of spontaneous colitis [38]. Muc2 deficiency represents a fundamental compromise in barrier function which might be common in both human and mice. In fact, Muc2 deficient mice have been shown to be similar to active human UC disease [39]. pDCs were found to be exacerbated in intestinal mucosa and mesenteric lymph nodes of IBD patients. However, though they were activated in terms of other markers like TNFα, CD40, IL6 and IL8, they were reduced in terms of their signature activation molecule such as IFNα [40]. Interestingly, their pathogenic property in acute DSS colitis was also found to be independent of Type 1 interferon signaling. Thus, intestinal pDCs are also a cell type with IBD translational opportunities.

While there is great therapeutic potential of these mononuclear phagocyte subsets in IBD in future, caution must be taken to interpret these data. Mononuclear phagocytes, unlike their effector lymphocyte counterparts (i.e. the T and B cells), are hyper sensitive to their milieu and might change course of action as a response. Though they correlate to a proinflammatory signature, the potential for utilizing them for inducing immunoregulation cannot be ruled out. DC2 has recently been found to be highly enriched in IBD colon samples in the context of anti-TNF treatment resistance [41]. We have mentioned earlier that DC2 but not DC1 express TGF-β activating integrin [37]. TGF-β activation can have several outcomes: either being pro pathological in the form of fibrosis or promoting Th17 and consecutively leading to inflammation or inducing Tregs and maintaining homeostasis. Thus, understanding the entire role of DC2 may require context-dependent assessment. On the other hand, DC1 and inflammatory monocytes were shown to occupy nodal positions in cellular network of healthy human and the network was significantly altered in ulcerative colitis [41]. In addition, inflammatory macrophages and activated DCs (CD86 + PDL1+) were found to be specifically enriched in inflamed ileum of Crohn’s disease patients [42]. Restoration of healthy network of cellular interaction may thus be envisaged by deeper investigation of cells like DC1, activated DC and inflammatory monocytes in IBD. Further understanding of their biology is required to target these inflammation-associated cells effectively. It’s worth to note that we also found that plasmacytoid DC-like subsets DC6 and DC5 correlate with inflammatory signature to the least extent. It suggests a lesser potential for a pathologic role of these subsets. Last but not the least, mononuclear phagocyte subsets are also known for inducing tolerance and can be targeted with an agonist approach to enhance their activity in gut homeostasis [43]. Thus, therapeutic targeting of mononuclear phagocyte may be subset and context specific, which offers the benefit of enhancing our chances of targeting the most pathologically committed subsets.

There are limitations in our analysis since our immune cell deconvolution analysis is based on xCell [25], which covers 31 immune cells and do not include Th17 or ILCs. Th17 cells or ILCs are reported to be enriched in inflamed colon and emerge as novel immune cell populations that play important roles in IBD pathogenesis [44, 45]. Gene expression of IL17 and IL22, key cytokines produced by Th17 and/or ILCs and linked to IBD pathogenesis, were further examined in treatment response data sets (Additional file 1: Figure S4). Both IL17A and IL22 are elevated significantly in inflamed tissues from CD or UC patients, before treatment. After treatment, both genes return to normal control level in responders, but not in non-responders. In addition, either IL17 or IL22 gene expression is higher in non-responders vs. responders in pre-treatment samples from infliximab studies, which suggest that potentially Th17 or ILC could contribute to treatment resistance. Interestingly, similar pattern was not observed in the vedolizumab samples.

We note that gene signatures used in this analysis were mostly derived from cells sorted from blood or lymphoid organs and may not represent the same cell type or state in the inflamed gut. In addition, gene signatures included are not possible to represent their diverse states which are potentially linked to different functions. In our analysis, we found γδ Τ cells are highly enriched in different inflamed colon locations, as well as in non-responders to infliximab treatment. Checking the original data source for this cell type signature, we found that those cells were sorted from human blood, which is comprised mostly of the Vδ2 subset of 훾δ T Cells. The tissue resident colonic 훾δ T cells are predominantly the Vδ1 subset [46]. Recently it has been demonstrated that Vδ1 subset is diminished and Vδ2 subset is enhanced in IBD especially in long-standing IBD who are non-responders to several therapeutic regimens [47]. Moreover, we found Tregs were highly enriched in inflamed tissues (Fig. 1a) and remained high in non-responder after infliximab treatment (Fig. 3). This may reflect an impaired Treg function. Human Tregs in IBD are reported to have a loss of ex vivo suppression function [48, 49], which have been recently demonstrated with FOXP3 + IL10+ Tregs being positive for TNFα and enhanced in inflamed compared to non-inflamed tissue in UC patients [41]. Impairment in Treg functionality is an important aspect of immune dysfunction associated with moderate to severe IBD patients. One can attempt to understand how these MNP subsets contribute to this important aspect by studying their proinflammatory cytokines like IL23, IL12, IL1B which are included in our proinflammatory signature and show high correlation with most subsets (Fig. 6). Thus, the significantly correlated proinflammatory signature in MNPs can potentially have a causal effect on direct impairment of Tregs by inducing TNFα like pro-inflammatory genes in Tregs. Advances in scRNA-seq technology provides an more unbiased workflow that permits sequencing of cells without prior knowledge of genes of interest and cells can be grouped based on their transcriptional signatures [50]. Applying this technology will allow us to more comprehensively analyze the immune system and tissue microenvironment. Additionally, it will enable us to discover novel cell subsets and states and to characterize their intrinsic biological functions and underlying signaling pathways involved in disease or treatments response vs. resistance.

The UC/CD data sets which we have deconvoluted here are from “mucosal biopsies” of patients with undisclosed depth. However, considering that the epithelial layer is definitely part of these mucosal biopsies, it is interesting to note that so much of MNPs are being identified. This is consistent to our data in mouse (Fig. 8) where we find these MNPs are drastically enhanced in IEL fraction. Considering the intrinsic function and epithelial location of these MNPs as direct and early immune sensors for damaged epithelium post infection/injury, this enrichment is not surprising. Recent studies suggest that intestinal epithelium might play a major role in the development and perturbation of IBD, which is considered as a translator between the microbiota and the immune system [51]. In addition, intestinal stromal cells are also reported to be a major player in mucosal immunity and homeostasis [31]. Research is needed to investigate the interplay between enriched immune cells, along with their adjacent cells, which could potentially reveal the development mechanisms of disease and provide novel therapeutic insights. For the potential interactions of enriched MNPs with epithelial and stromal cells, our chemokine expression evaluation is such an attempt. The result supports the potential role of mononuclear phagocytes in colon inflammation and treatment response, as well as implies their potential interactions with the intestinal environment cues. Moreover, scRNA-seq data from patient colonic mesenchymal cells have helped us to delineate the potential interaction with stromal cells (Additional file 1: Figure S3). Future data, some of them are emerging [41, 42], such as scRNA-seq profiling of the epithelial layer and colon biopsies, would be a more comprehensive approach to delineate the MNPs/lymphocytes/epithelial/stromal cells landscape in IBD.

## Conclusions

Our gene signature based in silico analysis showed elevated immune cells, especially mononuclear phagocytes in inflamed colon mucosa biopsies. Distinct cell type patterns were observed in patients with response vs. resistance to current biological therapies. Moreover, we found chemokines that are involved in mononuclear phagocytes trafficking and function are highly up-regulated in inflamed colon, and in treatment resistance samples. In addition to in silico observations, we also evaluated the levels of several macrophage and DC subsets; and confirmed their elevated abundance in a chronic DSS colitis model. Overall, the accumulation of mononuclear phagocytes in colitis tissue is an important event and need to be carefully teased out for proinflammatory or tolerogenic activity to understand their exact role in pathogenesis of IBD.

## Methods

### Transcriptomic data sets

Three transcriptomic studies (GSE100833, GSE73661, GSE16879) for samples from both CD and UC patients, representing different locations of the gut, disease severity and treatment response (infliximab, vedolizumab), were downloaded from GEO database and used in the analysis. For each data set, we standardized the transcriptome data across patients by quantile-normalization.

scRNA-seq data for stroma cells from UC patients were downloaded (GSE114374). Cells were QC, selected and normalized by using Seurat package (v2.3) [52].

From GSE16879, two separate data sets represent infliximab treatment study in CD and UC. In CD patients, there are 12 responders vs. 7 non-responders. In UC patients, there are 8 responders vs. 16 non-responders. From GSE73661, one study contains infliximab treatment in UC patients including 8 responders vs. 15 non-responders; the other study contains vedolizumab treatment in UC patients including data from 16 responders vs. 25 non-responders before treatment at week 0; and 13 responders vs. 6 non-responders after treatment at week 52. Detailed information regarding patient and biopsy can be found in [53, 54].

### Cell type signature and enrichment analysis

Immune cell type gene signatures were from xCell study which were compiled from over thousands of sorted human cell type transcriptomes from various resources [25]. There are 165 gene signatures with number of gene > = 15, representing a total of 31 immune cell types which were included in the analysis (iDC and NKT gene signatures were removed due to small number of genes).

DC, monocyte subset gene signatures are from scRNA-seq study of human blood DC, monocytes [28].

Two sample population GSEA analysis [26] was performed for each gene signature. Normalized enrichment score and *p* values were averaged for the same cell type.

Single sample enrichment analysis were based on the analysis of xCell [25].

### Correlation analysis of DC, monocyte subsets and proinflammatory signature

Spearman correlation analysis was performed for each pair of DC, monocyte subset (after removing common genes) for their expressions in either UC or CD transcriptomic data using R v3.5.1. The heatmaps were generated using gplots v3.0.1.1 (https://cran.r-project.org/web/packages/gplots/index.html) with default parameters. Similarly, their potential function in inflammation was also assessed by evaluating their correlation with a proinflammatory gene signature. The expression value of each signature was represented by the median value of all member in the specific data set.

### Performance comparison of predicting treatment non-response

Response prediction performance for each classifier (cell type) was implemented in ROCR package. The predicted value of each gene signature was the median value of all members in the data set. For cell type with multiple gene signatures, the final AUC score is the average of individual ones.

### Chronic DSS colitis models and tissue processing for cell analysis

Female Bl/6 J mice of around 7 weeks of age were purchased from Jackson Laboratories and housed in the animal facilities of Takeda California and maintained under regular specific pathogen free condition. Following 1 week of acclimatization, for some mice, water was replaced with 3% DSS for 3 cycles, each cycle consisting of 5 days. We performed in vivo experiment with 3 untreated and 6 DSS treated mice. All the 9 animals were the same gender, same date of birth and arrived at the animal facility at the same time and house for similar time period. Animals were randomly picked and put into 3 cages of 3 mice per cage. Mean wt. at the start was 18.8 +/− 1.1 g. In between two consecutive cycles, these DSS-fed mice were put on regular water for 15–20 days. All three DSS cycle were initiated before noon. Body weights were monitored on a regular basis. No special husbandry condition was employed. All mice were sacrificed on day 55 after the start of the first round of DSS by using CO2 based euthanasia as per our animal facility regulations. DSS treatment was performed in parallel i.e. two bottles were placed in two cages. While sacrificing for cell isolation, experimenters were blinded of the order of sacrifice. No adverse events were observed on course of the experiment. The colons of mice were removed from MLNs and other mesenteric structures, cut open longitudinally and fecal matters removed. Then the opened colon was transversely cut into small pieces of approximately 1 cm in length and intraepithelial and lamina propria fractions were extracted following protocol supplied by the manufacturer (LP dissociation kit, Miltenyi Biotec) with a few modifications. Briefly, small pieces of colon were incubated in pre-digestion solution (HBSS buffer containing 10 mM Hepes buffer, 5 mM EDTA, 5% FBS and 1 mM DTT) and continuously but gently shaken using a MACSmix Tube Rotator at 37 °C. This step was repeated for 2 rounds. After each round, tubes the tissue in pre-digestion solution were vigorously shaken for approximately 10 s and the content was passed through 70 μM filter to get the IEL fraction. Following two rounds of pre-digestion solution, the remaining tissue was washed in HBSS buffer containing only 10 mM Hepes buffer (passing through 70 μM filter to add to the IEL fraction) to remove the EDTA. Thereafter, the colon pieces were incubated in digestion solution (HBSS with Ca2++ and Mg2++ buffer containing 10 mM Hepes buffer, 5% FBS and requisite amount of enzymes provided in the kit) and gently shaken using a Max rotor at 37 °C. Finally, tissue pieces were disintegrated using appropriate program of the gentle MACS dissociator and passed through 70 μM filter to get the LP fraction. The whole experiment was repeated as a part of a different experiment which was not part of the current project.

All experiments were ethically performed according to our institutional guidelines. We are an AAALAC accredited organization using The Guide for the Care and Use of Laboratory Animals (Guide) as the primary standard for our animal care and use program. We are also a registered research facility with the California Department of Health Services and hold a Certificate of Approval to Keep and Use Laboratory Animals.

### Flow Cytometry

Single cell suspensions from IEL and LP were labelled with a cocktail of flurochrome conjugated antibodies against surface antigens purchased from either BD Biosciences (San Jose, CA) or Biolegend (San Diego, CA): anti-CD45 FITC, anti-CD19 PerCP Cy5.5, anti-Siglec H Pacific Blue, anti-I/A I/E BV605, anti-CD11b BV 650, anti-CD3e BV786, anti-B220 BUV496, anti-EpCam APC, anti-F4/80 AF700, anti-Ly6c APC-Cy7, anti-CD11c PE-CF594, anti-CD103 PE-Cy7 and Zombie aqua kit. Cells were fixed with 1% PFA prior to acquisition in Flow Cytometry. Cells were acquired using a 18 color BD LSRFortessa (San Jose, CA) and analyzed with FlowJO software (Ashland, Oregon).

## Supplementary information

**Additional file 1: Figure S1.** DC, monocyte subset signatures predict treatment non-response. (a) ROC curves for the performance of signatures in predicting infliximab response among 24 UC samples (GSE16879). (b) The area under the ROC curve (AUC) for signatures in predicting infliximab response among 24 UC samples (GSE16879) in a. (c) ROC curves for the performance of signatures in predicting infliximab response among 19 CD samples (GSE16879). (d) AUC for signatures in predicting infliximab response among 19 CD samples (GSE16879) in c. (e) ROC curves for the performance of signatures in predicting infliximab response among 23 UC samples (GSE73661). (f) AUC for signatures in predicting infliximab response among 23 UC samples (GSE73661) in e. (g) ROC curves for the performance of signatures in predicting vedolizumab response among 41 UC samples (GSE73661). (h) AUC for signatures in predicting vedolizumab response among 41 UC samples (GSE73661) in g. **Figure S2.** Evaluation of chemokine expression in treatment response data sets. (a) Chemokines retain high expression in non-responders after treatment of infliximab (GSE16879). (b) Median expression of chemokines involved in myeloid cell trafficking in CD and UC patients in response to infliximab (GSE16879). (c) Median expression of chemokines involved in myeloid cell trafficking in UC patients in response to either infliximab or vedolizumab (GSE73661). IFX: infliximab; VDZ: vedolizumab. NR: non-responder; R: responder. B/Before: before treatment; A/After: after treatment. W0: week 0 before treatment; W4_W6: week 4–6 after treatment of infliximab; W52: week 52 after treatment of vedolizumab. * *P* value < 0.05, ** *P* value < 0.01. **Figure S3.** Expression of myeloid cell related chemokines in the stromal cells from UC patients and healthy controls (HC). **Figure S4.** Expressions of IL17A and IL22 in response to biological treatment in IBD patients. (a) CD and UC patients (GSE16879). (b) UC patients (GSE73661). IFX: infliximab; VDZ: vedolizumab. NR: non-responder; R: responder. Before: before treatment of infliximab; After: after treatment of infliximab. W0: week 0 before treatment; W4_W6: week 4–6 after treatment of infliximab; W52: week 52 after treatment of vedolizumab.

## Data Availability

The datasets used and/or analyzed during the current study are available from the corresponding author on reasonable request.

## Abbreviations

aDC: Activated Dendritic cells
CD: Crohn’s disease
cDC: Conventional Dendritic cells
DC: Dendritic cells
GSEA: Gene set enrichment analysis
IBD: Inflammatory bowel disease
iDC: Inflammatory Dendritic cells
pDC: Plasmacytoid Dendritic cells
UC: Ulcerative colitis
