# Supplementary material for: Subsets of mononuclear phagocytes are enriched in the inflamed colons of patients with IBD
Source: BMC Immunol. 2019 Nov 12;20:42. doi: 10.1186/s12865-019-0322-z (PMC6852755; doi:10.1186/s12865-019-0322-z)
Supplement: Supplementary file 1 — Additional file 1: Figure S1. DC, monocyte subset signatures predict treatment non-response. (a) ROC curves for the performance of signatures in predicting infliximab response among 24 UC samples (GSE16879). (b) The area under the ROC curve (AUC) for signatures in predicting infliximab response among 24 UC samples (GSE16879) in a. (c) ROC curves for the performance of signatures in predicting infliximab response among 19 CD samples (GSE16879). (d) AUC for signatures in predicting infliximab response among 19 CD samples (GSE16879) in c. (e) ROC curves for the performance of signatures in predicting infliximab response among 23 UC samples (GSE73661). (f) AUC for signatures in predicting infliximab response among 23 UC samples (GSE73661) in e. (g) ROC curves for the performance of signatures in predicting vedolizumab response among 41 UC samples (GSE73661). (h) AUC for signatures in predicting vedolizumab response among 41 UC samples (GSE73661) in g. Figure S2. Evaluation of chemokine expression in treatment response data sets. (a) Chemokines retain high expression in non-responders after treatment of infliximab (GSE16879). (b) Median expression of chemokines involved in myeloid cell trafficking in CD and UC patients in response to infliximab (GSE16879). (c) Median expression of chemokines involved in myeloid cell trafficking in UC patients in response to either infliximab or vedolizumab (GSE73661). IFX: infliximab; VDZ: vedolizumab. NR: non-responder; R: responder. B/Before: before treatment; A/After: after treatment. W0: week 0 before treatment; W4_W6: week 4–6 after treatment of infliximab; W52: week 52 after treatment of vedolizumab. * P value < 0.05, ** P value < 0.01. Figure S3. Expression of myeloid cell related chemokines in the stromal cells from UC patients and healthy controls (HC). Figure S4. Expressions of IL17A and IL22 in response to biological treatment in IBD patients. (a) CD and UC patients (GSE16879). (b) UC patients (GSE73661). IFX: infliximab; VDZ: [file 12865_2019_322_MOESM1_ESM.ppt]

## Slide 1
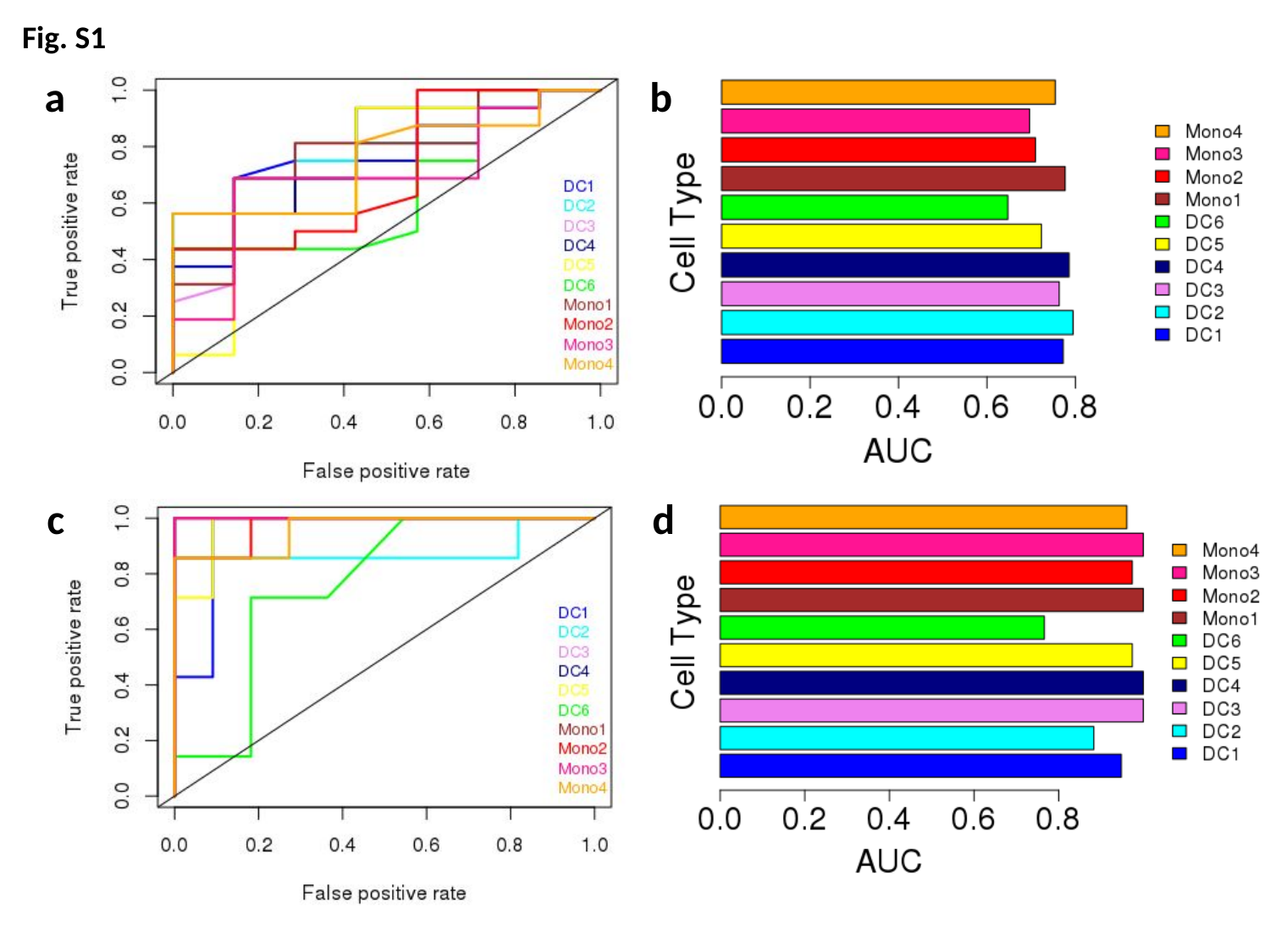

Fig. S1
a
b
c
d

## Slide 2
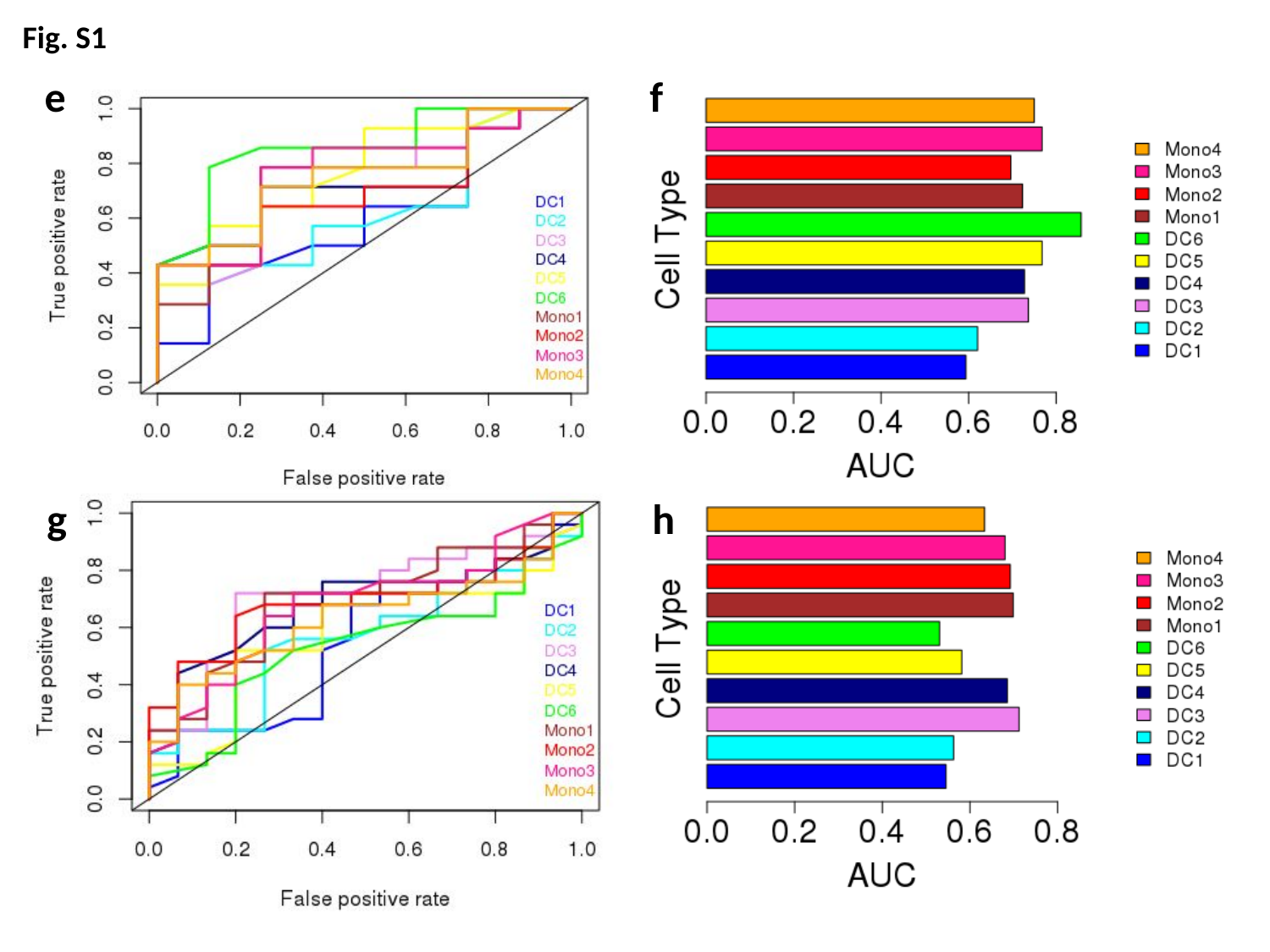

Fig. S1
e
f
g
h

## Slide 3
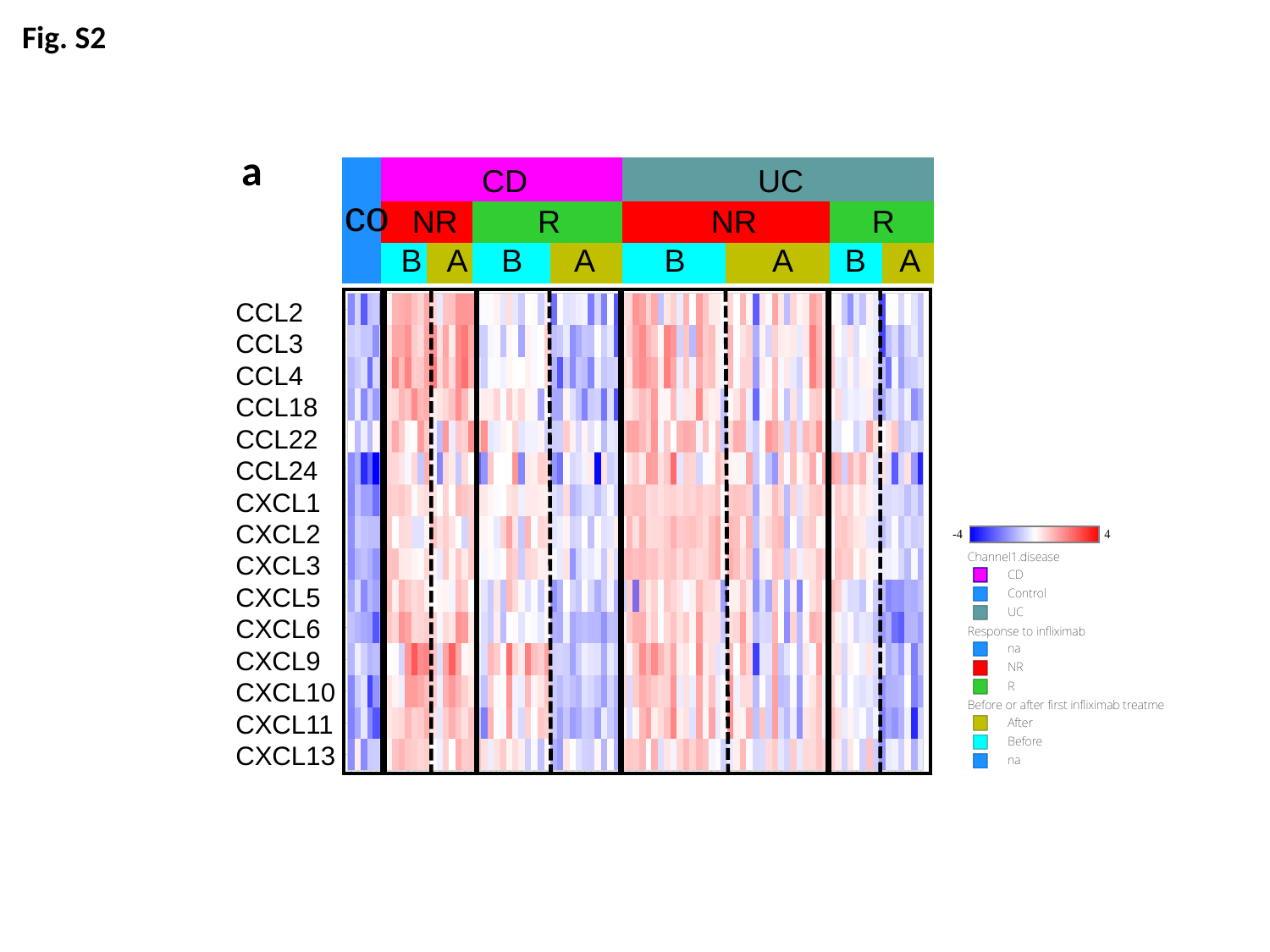

Fig. S2
a
CD UC
co
 NR R NR R
B A B A B A B A
CCL2
CCL3
CCL4
CCL18
CCL22
CCL24
CXCL1
CXCL2
CXCL3
CXCL5
CXCL6
CXCL9
CXCL10
CXCL11
CXCL13

## Slide 4
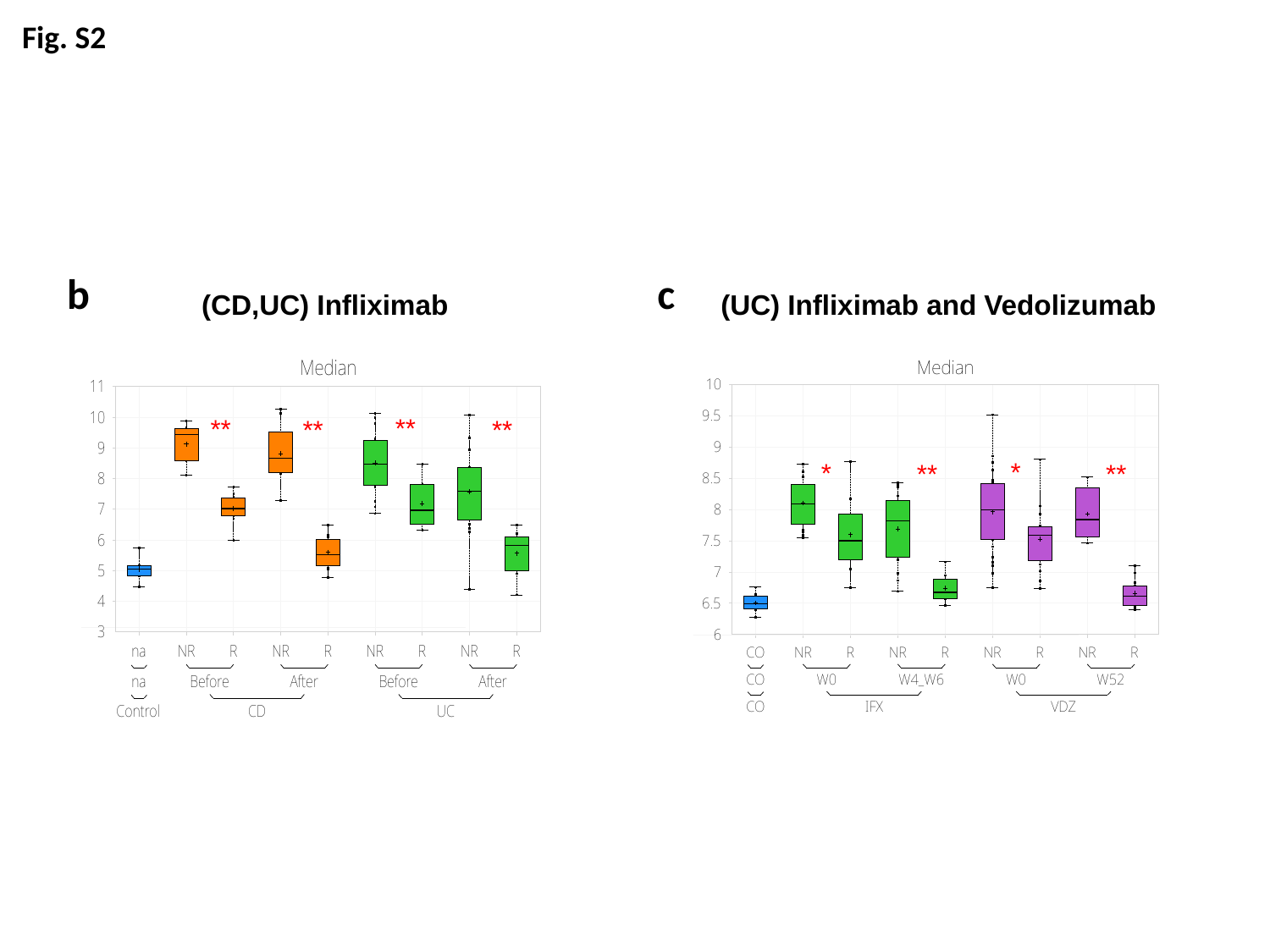

Fig. S2
b
c
(CD,UC) Infliximab
(UC) Infliximab and Vedolizumab
**
**
**
**
*
*
**
**

## Slide 5
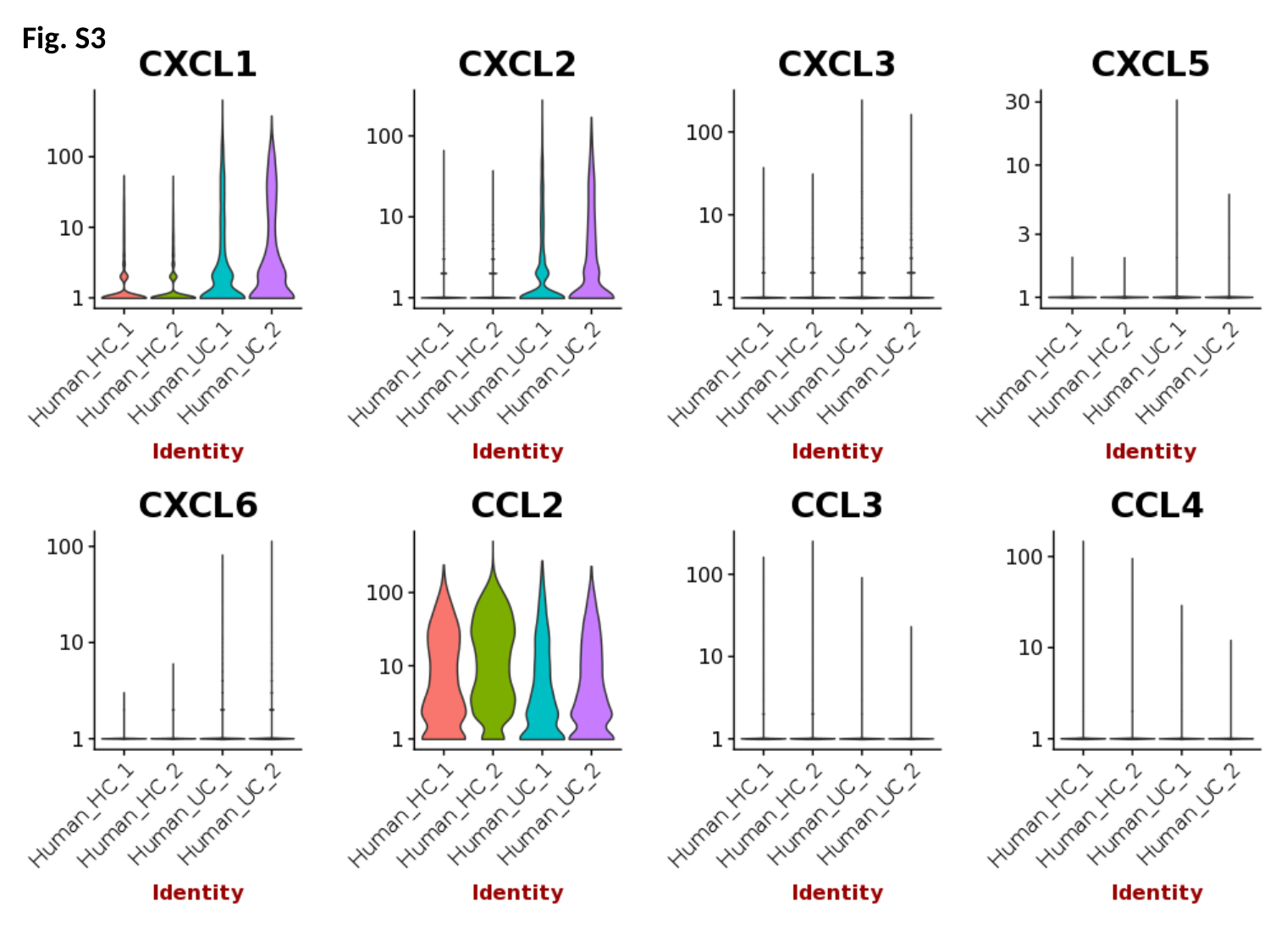

Fig. S3

## Slide 6
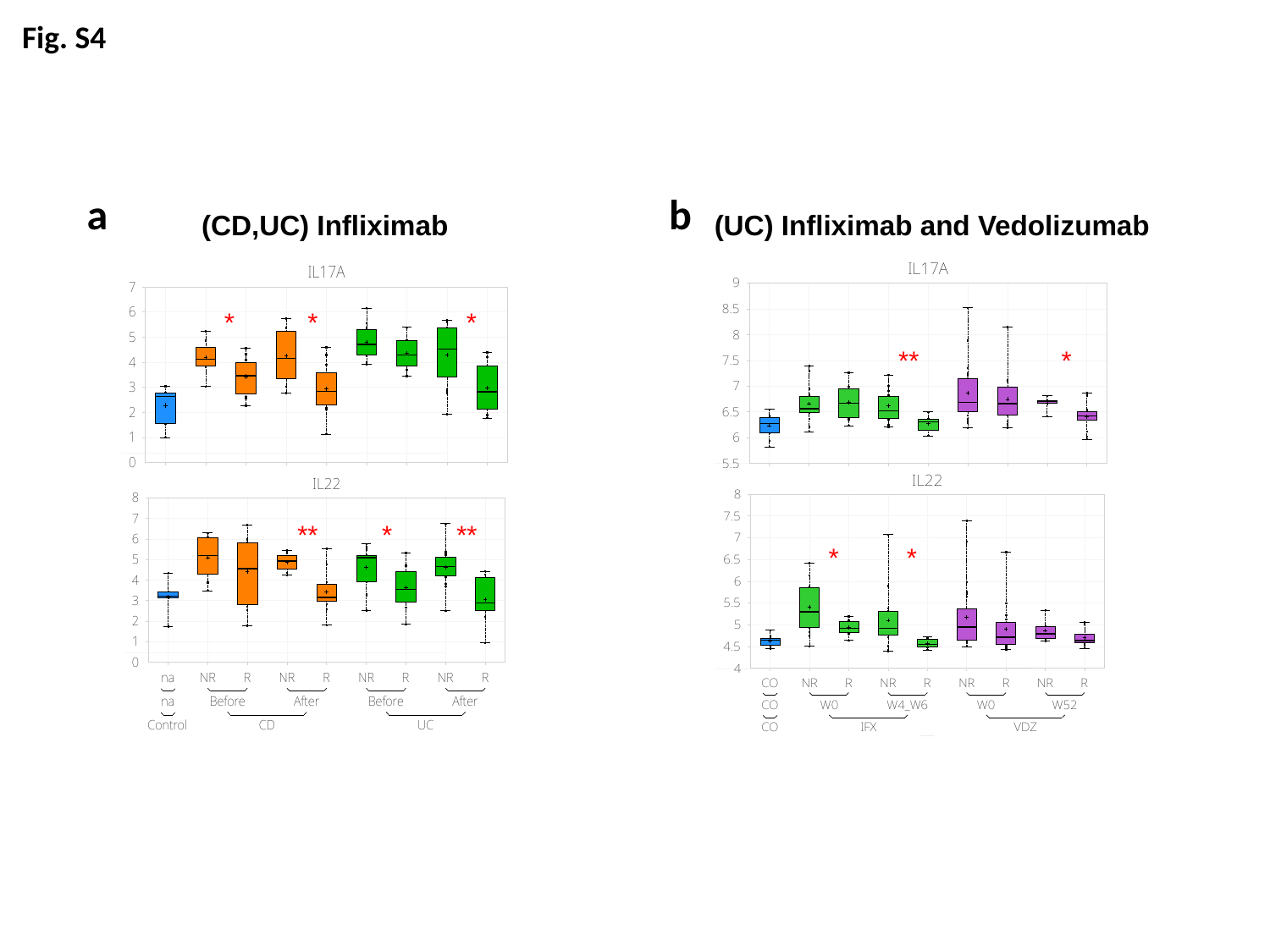

Fig. S4
a
b
(CD,UC) Infliximab
(UC) Infliximab and Vedolizumab
*
*
*
**
*
**
*
**
*
*
